# *Eugenia sulcata* (Myrtaceae) Nanoemulsion Enhances the Inhibitory Activity of the Essential Oil on P2X7R and Inflammatory Response In Vivo

**DOI:** 10.3390/pharmaceutics14050911

**Published:** 2022-04-21

**Authors:** Bettina Quintanilha Magalhães, Francisco P. Machado, Paola S. Sanches, Bárbara Lima, Deborah Quintanilha Falcão, Natalia von Ranke, Murilo Lamim Bello, Carlos Rangel Rodrigues, Marcelo Guerra Santos, Leandro Rocha, Robson X. Faria

**Affiliations:** 1 Laboratório de Biotecnologia, Alimentação e Saúde, Universidade Federal Fluminense, R. Alexandre Moura, 8-São Domingos, Niterói 24210-200, Brazil; betinamagalhaes@id.uff.br; 2 Laboratório de Tecnologia de Produtos Naturais, Faculdade de Farmácia, Universidade Federal Fluminense, Rua Doutor Mário Viana 523, Santa Rosa, Niterói 24241-000, Brazil; fmachado@id.uff.br (F.P.M.); paolasanches@id.uff.br (P.S.S.); bglimafarm@gmail.com (B.L.); lean.machado@gmail.com (L.R.); 3 Post-Graduation Program in Vegetal Biotechnology and Bioprocesses, Rio de Janeiro Federal University, Avenida Carlos Chagas Filho, 373, Cidade Universitária, Niterói 21941-599, Brazil; 4 Laboratório de Tecnologia Farmacêutica I, Faculdade de Farmácia, Universidade Federal Fluminense, Rua Doutor Mário Viana 523, Santa Rosa, Niterói 24241-000, Brazil; deborah@vm.uff.br; 5 Faculdade de Farmácia, Universidade Federal do Rio de Janeiro, Rio de Janeiro 21941-902, Brazil; nataliavonranke@outlook.com (N.v.R.); murilolamim@pharma.ufrj.br (M.L.B.); rangelfarmacia@gmail.com (C.R.R.); 6 Departamento de Ciências, Faculdade de Formação de Professores, Universidade do Estado do Rio de Janeiro, Francisco Portela 1470, São Gonçalo 24435-000, Brazil; marceloguerrasantos@gmail.com; 7 Laboratório de Avaliação e Promoção da Saúde Ambiental, Instituto Oswaldo Cruz, Avenida Brasil 4365, Manguinhos, Rio de Janeiro 21040-900, Brazil; 8 Programa de Pós-graduação em Ciências e Biotecnologia, Universidade Federal Fluminense, R. Alexandre Moura, 8-São Domingos, Niterói 24210-200, Brazil

**Keywords:** ATP, Myrtaceae, cytokines, ionic channels, nanoemulsion, *Eugenia sulcata*

## Abstract

P2X7R is a purinergic receptor with broad expression throughout the body, especially in immune system cells. P2X7R activation causes inflammatory mediators to release, including interleukin-1β (IL-1β), the processing and release of which are critically dependent on this ion channel activation. P2X7R’s therapeutic potential augments the discovery of new antagonistic compounds. Thus, we investigated whether the *Eugenia sulcata* essential oil could block P2X7R activity. The essential oil (ESO) dose-dependently inhibited ATP-promoted PI uptake and IL-1β release with an IC_50_ of 113.3 ± 3.7 ng/mL and 274 ± 91 ng/mL, respectively, and the essential oil nanoemulsion (ESON) improved the ESO inhibitory effect with an IC_50_ of 81.4 ± 7.2 ng/mL and 62 ± 2 ng/mL, respectively. ESO and ESON reversed the carrageenan-activated peritonitis in mice, and ESON exhibited an efficacy higher than ESO. The majority substance from essential oil, β-caryophyllene, impaired the ATP-evoked PI uptake and IL-1β release with an IC_50_ value of 26 ± 0.007 ng/mL and 97 ± 0.012 ng/mL, respectively. Additionally, β-caryophyllene reduced carrageenan-induced peritonitis, and the molecular modeling and computational simulation predicted the intermolecular interactions in the P2X7R situs. In silico, results indicated β-caryophyllene as a potent allosteric P2X7R antagonist, although this substance may present toxic effects for humans. These data confirm the nanoemulsion of essential oil from *E. sulcata* as a promisor biotechnology strategy for impaired P2X7R functions and the inflammatory response.

## 1. Introduction

The Myrtaceae family is represented in Brazil by 24 genera and 927 species, of which 707 are endemic [1]. This family is highly pertinent in Brazil and symbolizes a dominant woody family from Mata Atlantica. One synapomorphy found in this family is the leaves with spherical secretory cavities containing terpenoids and other aromatic, spicy resinous compounds [1,2]. Eugenia represents the largest genera in the Myrtaceae family. This genus possesses Brazil with 356 species in different habitats, and 274 are endemic [2]. In addition, several species have folk use in the coastal population [3,4,5]. In general, the essential oil composition from *Eugenia* spp. leaves has a predominance of cyclic sesquiterpenes, and monoterpenes appear as minor constituents [6].

The species *Eugenia sulcata* Spring ex Mart. possess edible fruits used for preparing jams or juices and are commonly known as “murtinha”, “murta preta”, and “pitangueira selvage” [5,7]. Although the *E. sulcata* effect is not well described, this plant is commonly used as an anti-diarrheic and for fever medical care [7]. In addition, in vitro subjects indicated the anticholinesterase effect of the essential oil [8]. Therefore, there is insufficient knowledge about their action mechanism to operate these biological reactions, specifically the antipyretic effect.

The P2X7 receptor (P2X7R) is atypical because when stimulated by a low micromolar ATP concentration (<100 µM), it activates an ionic channel (cut off in turn of 150 Da) and, when evoked by a high micromolar ATP concentration (>100 µM), activates a large conductance pore (cut off in turn of 900 Da). Currently, we do not know the exact mechanism it uses to form this large conductance pore. Some scientific groups suggest a P2X7R cationic channel pore dilation and others, the activation of another protein responsible for the pore opening.

However, we understand that low conductance channel and pore functions are reduced due to the deficit of inhibitors to segregate between both entities. Additionally, ionic channel or pore activation stimulates IL-1β release, associated with a pro-inflammatory activity, fever evolution, and cell death. Our group recently checked and declared a plant extract from the Clusiaceae family with an inhibitory response on P2X7R pore formation [9]. Therefore, the need for the examination of a new P2X7R antagonist has currently increased considering their involvement in diseases [10,11,12,13], although scarce scientific groups have supposed substances as selective for inhibiting the low conductance channel or pore. Additionally, we investigated the inhibitory response of *E. sulcata* essential oils (ESO) from Restinga of Jurubatiba against the P2X7R channel and pore functions in murine peritoneal macrophages (MPM). Following the results, the ESO potently inhibited P2X7R pore formation.

In contrast, this essential oil was a poor blocker of the cationic channel activity. Furthermore, the essential oil inhibited inflammation in mice after intraperitoneal and oral administration. Therefore, we formulated and tested an essential oil nanoformulation (ESON) to improve oil dispersibility in an aqueous medium and the inhibitory reaction on P2X7R. 

## 2. Materials and Methods

### 2.1. Plant Material

Leaves of *Eugenia sulcata* Spring ex Mart. were collected in Restinga de Jurubatiba National Park, Rio de Janeiro State (22°12′98.4″ S–41°35′00.8″ W), Brazil, during the day on 8 January 2019. Sisbio/ICMbio (13659-14) and SisGen (A0D648D) authorized the plant material research and collection. The botanist, Dr. Marcelo Guerra Santos, identified the species and a voucher specimen was deposited at the herbarium of the Faculdade de Formação de Professores (Universidade do Estado do Rio de Janeiro, Brazil) under register number RFFP 13.788.

### 2.2. Extraction of the Essential Oil and Chemical Characterization

Fresh leaves (4.950 g) were turbolized with distilled water. Then, this material was placed in a 5 L bottom flask and submitted to hydrodistillation for 4 h using a Clevenger apparatus. The essential oil was collected, dried over anhydrous sodium sulfate, and stored at 4 °C for further analysis.

The *E. sulcata* essential oil (ESO) was inspected using a GC-MSQP2010 (Shimadzu, Kyoto, Japan) gas chromatograph, supplied with a mass spectrometer, and a GC-2014 (Shimadzu, Kyoto, Japan) gas chromatograph provided with a flame ionization detector (FID).

The GC-MS background was as follows: the temperature of injection was 260 °C, the helium gas followed a flow rate of 1 mL/minute and a split injection with a rupture ratio of 1:40. The initial oven temperature was 60 °C and then rose to 290 °C at a rate of 3 °C/minute. ESO (1 µL) solubilized in dichloromethane (1:100) was introduced into an RTX-5 column (0.25 mm ID, 30 m in length, 0.25 μm and film thickness). Mass spectrometry (MS) electron ionization was 70 eV, and the rate was 1 scan/s.

GC-FID adjustments comprised an injector with temperature of 260 °C, gas helium with flow rate of 1 mL/minute, and split injection with a ratio of 1:40. The initial oven temperature was 60 °C and elevated to 290 °C at a rate of 3 °C/min. ESO (1 µL) dissolved in dichloromethane (1:100) was inserted into an RTX-5 column (0.25 mm ID, 30 m in length, 0.25 μm in film thickness). The flame ionization detector (FID) temperature was 290 °C.

We compared the Arithmetic Index (AI) for the identification of compounds using the retention times of a mixture of a series of aliphatic hydrocarbons (C9–C30) [14]. The MS fragmentation design of compounds was correlated with NIST mass spectrum libraries. The relative chemical composition abundance was determined by flame ionization gas chromatography (CG-FID) under GC-MS circumstances. FID peak area normalization method was employed to analyze the substance percentages [15].

### 2.3. Nanoemulsion Preparation and Characterization

The essential oil nanoemulsions were obtained by the phase inversion temperature method. First, the oil phase, containing the ESO and surfactant blend, was heated to 40 ± 1 °C. Next, the aqueous phase was heated to 70 ± 5 °C and then slowly dripped over the oil phase under magnetic stirring at 400 ppm for 10 min, followed by a water bath at room temperature for another 10 min [16,17]. Nanoemulsion characterization was realized by the dynamic lightning scattering technique (DLS) in a nanosizer (Malvern, UK), using the droplet size (nm) and polydispersity index as parameters. Initially, ten formulations with 90% (*w*/*w*) of water, 5% (*w*/*w*) of ESO, and 5% surfactans blend were prepared to establish the proportions of the surfactants (polysorbate 20 and sorbitan monooleate 80) and determine the hydrophilic–lipophilic balance (HLB) of the ESO with range values between 12 and 16.7. Then, the formulation optimization established an excellent HLB value. The ESO amount (2.5–7.5% *w*/*w*), the mixture of surfactants (2.5–7.5% *w*/*w*), and water (82.5–92.5% *w*/*w*) of the *E. sulcata* essential oil nanoemulsion (ESON) varied to determine the optimal nanoemulsion formulation.

### 2.4. Mice Peritoneal Macrophages

We inoculated 10 mL of RPMI-1640 medium into the peritoneal cavity of male Swiss mice to harvest mouse peritoneal macrophages (MPM). The collected cells were centrifuged, resuspended, and plated in microplate wells with aliquots (0.5 mL) of cell suspension. These cells were incubated for 1 h in a humidified atmosphere (37 °C, 5% CO_2_) for cell adhesion. We removed the nonadherent cells after washing with RPMI-1640 medium. Firmly adhering cells were maintained in RPMI medium with 10% fetal bovine serum (FBS) and gentamycin (1 μL/mL) and used for the experimental assay as described previously [18].

### 2.5. LDH Release Assay

In the cytotoxicity assay, after treatment with ESO, ESON or β-caryophyllene, the presence of LDH in the supernatant media was measured using a cytotoxicity detection kit (Sigma kit for LDH) fin according to the manufacturer’s instructions.

### 2.6. Intracellular Ca^2+^ Measurements

Cytofluorometric analysis of Ca^2+^ uptake. Fluo-3, a Ca^2+^-sensitive indicator, was used to monitor the changes in intracellular Ca^2+^ [19]. The baseline was established with untreated cells for 1 h and loaded with 1 mM FLUO-3 plus 100 μM verapamil at 37 °C for 20 min in extracellular saline. Immediately after that, the changes in fluorescence were determined in 1 × 10^6^ loaded cells over 60 s by flow cytometry (FACScan Calibur). Cytometric analysis was done by Summit software. Cultures were either untreated (as described above); macrophages stimulated for 5 min with 1 mM ATP alone; preincubated with 100 nM BBG or doses of the essential oils for 10 min followed by stimulation with 1 mM ATP or treated with Triton × 100 (0.01%) as a positive control.

### 2.7. Dye Uptake Assay

Cell permeabilization was visualized by the differential uptake of propidium iodide (PI) (696 Da). MPM was incubated with 1 mM ATP in the presence or absence of ESO or P2X7R antagonists for 25 min at 37 °C. ESO and P2X7R antagonists were bred for 10 min before 1 mM ATP prescription or not. ESO and ESON doses ranged from 0.1 ng/mL to 25 µg/mL. P2X7R inhibitors were used at a concentration of 100 nM. Propidium iodide (0.05 mg/mL in PBS) was added in the last 5 min of incubation. Ninety-six-well microplates were washed with saline solution (150 mM KCl, 5 mM NaCl, 1 mM MgCl_2_, 0.1 mM EGTA, and 10 mM HEPES, pH 7.4) or PBS, pH 7.4 and observed under a plate reader (Espectramax M5). The dye was excited at 488 nm, and the fluorescence emission was observed at 560 nm or using a fluorescence microscope (Nikon) equipped with rhodamine (546/FT 580/LP 590) and fluorescein (450-490/FT 510/LP 520) filters.

The fluorescence pattern was analyzed by flow cytometry as described by Nihei (2000) and collaborates [20] in some analyses. Dead cells and cellular debris were excluded based on low forward and side scatters and the highest fluorescence profile. At the same time, 1 mM ATP in the presence or absence of ESO or P2X7R antagonists was incubated at 37 °C for 25 min. PI was added in the last 5 min and immediately analyzed by flow cytometry. The doses of ESO varied from 0.01 ng/mL to 10 µg/mL. P2X7R antagonists (100 nM BBG or KN-62 (data not shown)) were used as controls. 

### 2.8. Sodium Green Assay

Cells (1.0 × l0^6^ cells) were centrifuged and resuspended in 1 mL loading buffer containing 10 μM sodium green tetraacetate (Molecular Probes, Inc., Eugene, OR, USA) and incubated at 37 °C for 1 h, with gentle agitation every 15 min to prevent cells from settling to the bottom of the tubes.

Stock solutions of dye consisted of 5 mM sodium green tetraacetate or SBFI-AM dissolved in dimethyl sulfoxide (DMSO) and mixed with an equal volume of 10 μM verapamil (Molecular Probes, Inc., Eugene, OR, USA). Sodium green free acid (cell impermeant) added in several buffers with various Na^+^ concentrations ([Na ^+^] + [K ^+^] = 145 mM) and pH. MPM (1 × l0^6^ cells) incubated with sodium green tetraacetate were calibrated with gramicidin, and the dye was excited at 488 nm. Emission was measured at 500–600 nm. All recordings were performed on a FACS Calibur (Becton & Dickinson, Franklin Lakes, NJ, USA). Intracellular Na^+^ calibration was performed following Amorino and Fox, 1995 [21].

### 2.9. Oxide Nitric Release Assay

MPMs treated or not with ATP for 5 min, were preincubated or not with P2X7R antagonists, ESO and ESON for 5 min. We used the colorimetric assay using the Griess method to indirectly measure nitric oxide The nitrite standard reference curve was constructed in a 96-well plate containing sulfanilamide solution (1% sulfanilamide in 5% phosphoric acid, Sigma-Aldrich, St. Louis, MO, USA). Treated or standard wells received NED solution (0.1% N-1-naphthyl ethylene diamine dihydrochloride, Sigma-Aldrich, St. Louis, MO, USA, in water). The absorbance was quantified within 10 min at the absorbance of 550 nm (SpectraMax M5, Molecular Devices, San Jose, CA, USA).

### 2.10. Spectrofluorometric Measurement of ROS

Reactive oxygen species (ROS) generation was evaluated using 2′,7′-dichlorofluorescin diacetate (DCFH2-DA) [22]. Peritoneal macrophages (5 × 10^5^ cells/mL) were seeded in a 96-well plate suspended in extracellular saline and loaded for 15 min with 5 μM DCFH2-DA in a water bath at 37 °C with agitation. Unloaded dye was removed by centrifugation at 1500× *g* for 5 min. Then, the pellet was resuspended in extracellular saline without or with 1 mM CaCl_2_ and incubated at 37 °C for 5 min. Cells were stimulated with vehicle (control), ATP (1 mM), BBG (100 nM), and ESO (1 ng/mL–10 µg/mL) for 5 min at 37 °C, and then the fluorescence was measured using a spectrofluorometer with excitation and emission wavelengths of 501 and 522 nm, respectively (SpectraMax M5, Molecular Devices, San Jose, CA, USA). The calibration curve was obtained for serial DCF dilutions [23], to control by interpolation of the ROS amount produced by 5.0 × 10^5^ cells. Data are displayed as the mean ± SD.

### 2.11. IL-1β Enzyme-Linked Immunosorbent Assay

Differentiated MPMs, treated with 10 ng/mL interferon-γ (IFN-γ) for 24 h, were stimulated with *Escherichia coli* lipopolysaccharide (LPS, serotype 0127: B8) before ATP stimulation. P2X7R-mediated IL-1β and TNF-α release obtained from these cells was treated with 25 ng/mL LPS for 4 h and a second stimulus with ATP (5 mM) followed in the final 30 min of LPS treatment. P2X7 receptor antagonists, ESO, and ESON were preincubated 30 min before the ATP addition. IL-1β and TNF-α levels were determined in according to a standard kit (Bioscience, San Diego, CA, USA and Abcam, Cambridge Biomedical Campus, Cambridge, UK).

### 2.12. Electrophysiologic Measurements

Whole-cell configuration was realized as mentioned by Faria et al. [24]. No current compensation was adapted for currents less than 1500 pA. Above this level, the ionic currents were adjusted by 91%. When the series resistance substantially increased, the measurements were discarded. MPM cell capacitance (mean ± SD, 12.1 ± 3.03 pF; *n* = 63) was obtained after applying a 20-mV hyperpolarizing pulse from a holding potential of 20 mV. All subsequent adjustments were performed as previously described [24].

Patch-clamp experiments were carried out. All substances were solubilized in saline solution immediately before use. Ion currents were recorded after application of 10 μM (for 5 s) or 1 mM ATP (for 300 s) in the presence or absence of the P2X7R antagonists, ESO, ESON, and β-caryophyllene. All experiments were realized under perfusion (RC-24 chamber, Warner Instrument Corp, Holliston, MA, USA) at a rate of 1 mL/minutes.

### 2.13. In Silico Assays

Three-dimensional structures of the diterpenes were built and optimized using SPARTAN’10 software as described in previous works [25,26].

We evaluated the hypothetical β-caryophyllene interaction with the P2X7R using two blinded molecular dockings. β-caryophyllene was inserted as the ligand, and another assay with the A740003 antagonist, was performed as the ligand. Docking with A740003 was used to validate the method and as a parameter to compare the β-caryophyllene results. Both blind molecular docking experiments were performed against the whole P2X7 structure.

The blind molecular docking of A740003 predicted most of its interaction into the allosteric site. As revealed in the Karasawa e Kawate (2016) work [27], the A740003 quinolone ring established hydrophobic interactions with Phe88 and Tyr298 residues of the dimethoxybenzene ring and established hydrophobic interactions with Tyr299.

The superposition of the A740003 conformation extracted from PDB ID: 5U1U and the most favorable conformation generated by blind molecular docking are shown in Supplementary Figure S1. The methods for molecular docking have been described in previous reports [25,26,28].

The prediction of the physicochemical and toxicological profiles was performed by ADMET Predictor^®^ (Simulation Plus, Lancaster, CA, USA).

### 2.14. In Vivo Assay

#### 2.14.1. Experimental Mice

Our experimental procedures followed the Ethical Principles in Animal Experimentation followed by the Brazilian College of Animal Experimentation and adopted by the FIOCRUZ Research Ethics Committee (number LW-5814 and LW-35/16). We used male mice (*Swiss Webster)* of 4–5 weeks provided by the Institute of Science and Technology in Biomodels/Fiocruz).

#### 2.14.2. Carrageenan-Induced Peritonitis

Male Swiss mice administered with intrathoracic (i.t.) inoculation of 0.5 mL of carrageenan (200 µg/cavity), or sterile saline (0.9%) (control group) using a needle (13 × 0.45 mm) delicately added at a depth of 1 mm into the right side of the peritoneal cavity.). In addition, dexamethasone (10 mg/kg), A740003 (10 mg/kg), ESO, and ESON were orally administered 1 h before carrageenan application. Treatment with carrageenan (200 µg/cavity) for 4 h was followed by animal euthanization with 10% ketamine hydrochloride and 2% xylazine hydrochloride. The peritoneal cavity was washed with 1 mL of heparinized saline (10 UI mL^−1^), and peritoneal wash aliquots were diluted in Turk solution (2% acetic acid) and counted in Neubauer chambers. A differential leucocyte study was performed using stained cytospins (Cytospin 3, Shandon Inc., Pittsburgh, PA, USA) by the May-Grünwald-Giemsa method. The cellular counts are reported as numbers of cells per cavity.

### 2.15. Statistical Analysis

Statistical correlations are represented as the mean ± SD (standard deviation), as designated in the text. One-way analysis of variance (ANOVA) was used to evaluate the statistical significance of divergences between means. After this analysis, we realized a Tukey’s test. A value of *p* < 0.05 was considered significant for bicaudal analysis.

## 3. Results

### 3.1. Essential Oil Composition from Eugenia sulcata

After the extraction, the essential oil from fresh leaves of *E. sulcata* presented a clear light-yellow aspect and yielded 1.08% (*w*/*w*). Investigations by GC-FID and GC-MS demonstrated that sesquiterpenes represented the largest fraction (75.6%), and β-caryophyllene was the principal substance, corresponding to 18.65 % (*w*/*w*), followed by δ-cadinene (10.0%), and α-pinene (6.75%). The remaining substances found in this essential oil are presented in Table 1.

### 3.2. Nanoemulsion Constitution

We prepared numerous nanoemulsion formulations of 5% *w*/*w* ESO, 5% surfactant (polysorbate 20 and sorbitan monooleate 80), and 90% water with hydrophilic–lipophilic balances (HLB) between 12 and 16.7. The smallest droplets diameter, determined by DLS, was recognized for the mixture with HLB 16.25, which was selected as the most promising one. Table 2 shows the proportions between surfactants (polysorbate 20 and sorbitan monooleate 80), the HLB values, and corresponding droplets size. The nanoemulsion with HLB of 16.25 presented lesser droplet size values of 132.83 ± 3.12. The selection criterion of the most promisor surfactants proportion was the smallest mean size (nm). In this context, the proportion of 96.37 % (*w*/*w*) of polysorbate 20 and 3.63 % (*w*/*w*) of sorbitan monooleate 80 were considered the most promisor to the ESO because fraction exhibited a short particle size (76.57 ± 4.32) and used the least quantity of surfactants in the formulation (5%). The emulsions between 15.5 to 16.7 HLB presented a bluish aspect. The other HLB values presented milky-white coloration.

Furthermore, the formulation F6 was considered the optimal formulation to the *E. sulcata* essential oil nanoemulsion (ESON), because F6 had a small molecule size (76.57 ± 4.32) and used the least quantity of surfactants in the formulation (5%), and 0.438 ± 0.02 of polydispersity index (PdI). The composition was 92.5% (*w*/*w*) of water, 2.5% (*w*/*w*) of ESO, and 5% (*w*/*w*) of a surfactants blend (96.37% polysorbate 20 and 3.63% sorbitan monooleate 80). The other formulations are described in Table 3.

### 3.3. Toxicity Examinations on Mice Peritoneal Macrophages

**Figure 1:**
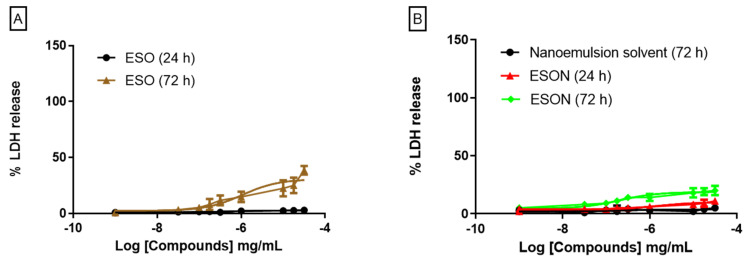
Cytotoxicity effect of ESO and ESON on mammalian cells evaluated by LDH release. (**A**) LDH release quantification after the stimulus with crescent ESO concentrations for 24 and 72 h; (**B**) LDH release quantification after the stimulus with crescent ESON concentrations for 24 and 72 h. Profiles are representative of 3–4 independent experiments for LDH release.

MPMs were stimulated with ESO and ESON concentrations ranging from 1 ng/mL to 70 µg/mL for 24 and 72 h. After 24 h, ESO and ESON did not cause effective LDH release in all tested concentrations (Figure 1A). In contrast, ESO treated for 72 h in concentrations higher than 1 μg/mL caused LDH release between 20–38%, when compared to treatment with the positive control, Triton-X 100 (Figure 1A).

ESON treatment for 72 h did not cause a toxic effect (Figure 1B), a factor related with essential oil solubility increases in the aqueous media. Therefore, the nanoemulsion system reversed the moderate toxicity caused for ESO in 72 h of stimulation.

### 3.4. ESO and ESON Effect on P2X7R Cationic Channel

**Figure 2:**
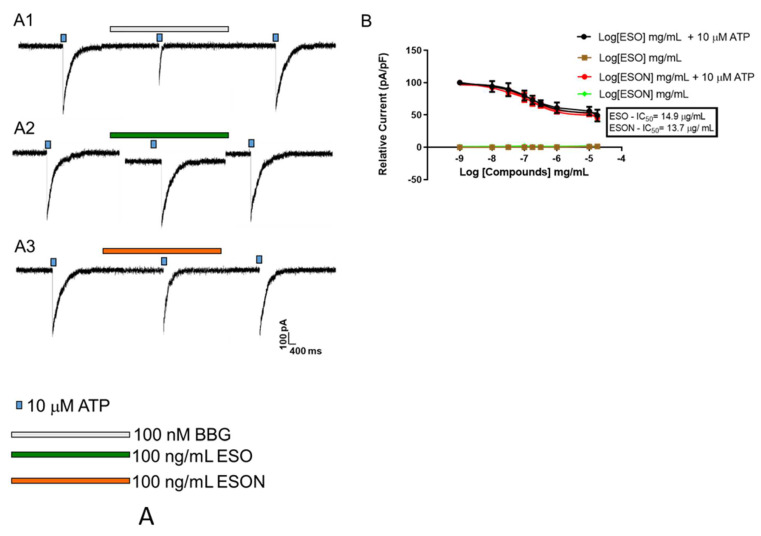
Dose–concentration curves for ESO and ESON inhibition on P2X7R cationic channel activity. (**A**) Whole-cell recordings of the cationic P2X7R activated by 10 µM ATP for 5 s at 30–37 °C on MPM. The reversibility of the inhibitory effect: a pulse of 10 µM ATP followed by perfusion of concentrations of ESO, ESON, or 100 nM BBG. After the washout, another pulse of 10 µM ATP was applied; (**B**) the plot represents the quantification of the data observed in (**A1**–**A3**); % relative current was recorded as a function of the ratio between the amplitude of the ionic current and cell capacitance. In all assays, we added the antagonists before ATP stimulation (1–10 min). Profiles are representative of 4 experiments for whole-cell recordings. *p* > 0.05 when compared to positive control.

ATP-induced P2X7R activation with ATP concentrations below 100 µM activates, primordially, preferentially low conductance cation channels permeable to organic cations of up to 100 Da, as NMDG (ref). Thus, we investigated the ESO inhibitory activity on ATP-induced cation channel through whole cell experiments. ATP induced a macroscopic current with amplitude in turn of 310 ± 46 pA (Figure 2A1–A3, first and third signals). The P2X7R antagonist, brilliant blue G (BBG), and ESO preincubated for 10 min before ATP stimulus inhibited P2X7R channel activity. BBG (100 nM) treatment effectively reduced ATP-induced current inhibition (220 ± 17 pA) compared with ESO (100 ng/mL) inhibition (90 ± 8 pA), Figure 2A1,A2, respectively, middle signal for both. ESO pretreatment in crescent concentrations exhibited an IC_50_ value of 14.9 ± 1.2 µg/mL (Figure 2B). ESON (100 ng/mL) produced an inhibitory effect (190 ± 11 pA) modestly higher than ESO (Figure 2A3, middle signal). ESON stimulation in crescent concentrations showed inhibitory activity with IC_50_ value similar to ESO (13.7 ± 2.5 pA), Figure 2B. ESO and ESON treatment alone did not induce ionic current interferences (Figure 2B).

### 3.5. ESO and ESON Effect on Intracellular Na^+^ and Ca^2+^ Mobilization, and ROS Production

**Figure 3:**
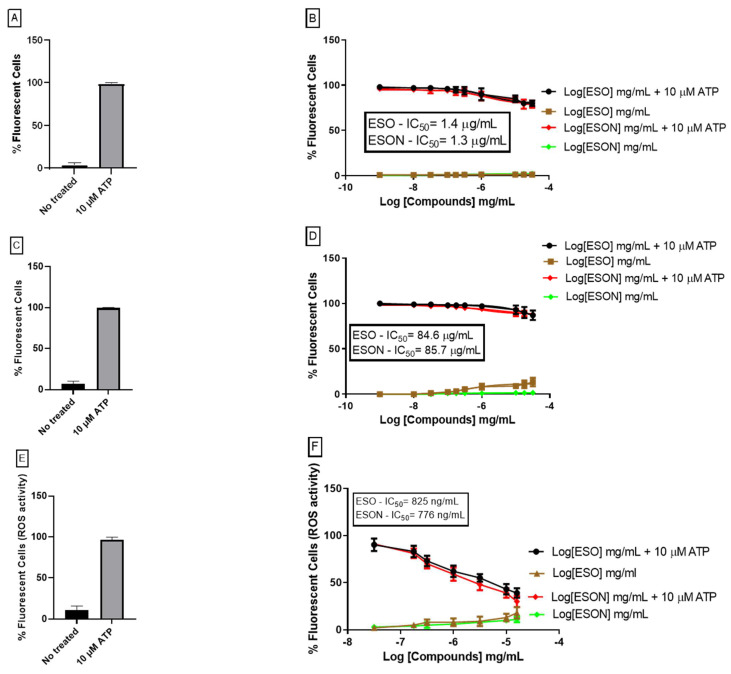
ESO and ESON effect on P2X7R activity. (**A**) No treated cells and cells treated with ATP analyzed with Sodium Green assay. (**B**) Dose–response curves of ESO and ESON with or without 10 µM ATP to detect changes in intracellular Na^+^ concentrations with Sodium Green assay. (**C**) No treated cells and cells treated with ATP analyzed with Fluo-3 assay. (**D**) Flow cytometry analyzed the dose–response curves of ESO and ESON with or without 10 µM ATP to detect changes in intracellular Ca^2+^ with Fluo-3 dye. (**E**) No treated cells and cells treated with ATP analyzed with DCFH2-DA dye. (**F**) Dose–response curves of ESO and ESON with or without 10 µM ATP analyzed by fluorescence plate reader to detect changes in intracellular ROS with DCFH2-DA dye; Values represent mean + SEM of total % ATP fluorescence effect. Profiles are representative of 3 separate experiments for Na^+^ and Ca^2+^ assay and 7 separate experiments for ROS assay.

Once P2X7R is preferentially permeable to cations such as Na^+^, K^+^ and Ca^2+^, this channel opening promotes the intracellular Na^+^ influx after treatment with 10 μM ATP for 5 min. ESO and ESON concentrations preincubated for 10 min before ATP application modestly reduced the Na^+^ influx analyzed by Sodium Green assay. We only observed substantial inhibition at concentrations above 500 ng/mL (Figure 3A). The IC_50_ value obtained for ESO and ESON inhibition was 1.4 µg/mL ± 0.02 and 1.3 ± 0.03 ^#^ µg/mL, (^#^ *p* > 0.05), respectively. The oil and nanoemulsion applied without subsequent ATP treatment did not cause an effect.

Another classical method of measuring the P2X7R cation channel function is to estimate the intracellular Ca^2+^ mobilization analyzed by FLUO-3 assay. The treatment with 10 μM ATP for 5 min preceded ESO and ESON concentrations for 10 min, similarly to Na^+^ influx, exhibited inhibition only in concentrations above 500 ng/mL (Figure 3B). The IC_50_ value obtained for ESO and ESON inhibition was 84.6 µg/mL ± 5.4 and 85.7 µg/mL ± 7 ^#^, (^#^ *p* > 0.05) (Figure 3B). The oil and nanoemulsion applied without subsequent ATP treatment did not cause an effect.

ATP-induced ROS production were measured on MPM and inspected by DCFH2-DA assay. Applying 10 mM ATP for 3 min led to a vast ROS production, and BBG reduced this effect (data not shown). ESO preincubated for 10 min reduced the ATP-induced ROS production. The IC_50_ value observed was 825 ng/mL ± 16 for ESO inhibition (Figure 3C) and 776 ng/mL ± 4 ^#^, (^#^ *p* > 0.05) for nanoemulsion inhibition. The oil and nanoemulsion applied without subsequent ATP treatment did not cause an effect.

### 3.6. ESO and ESON Activity on ATP-Induced Uptake

**Figure 4:**
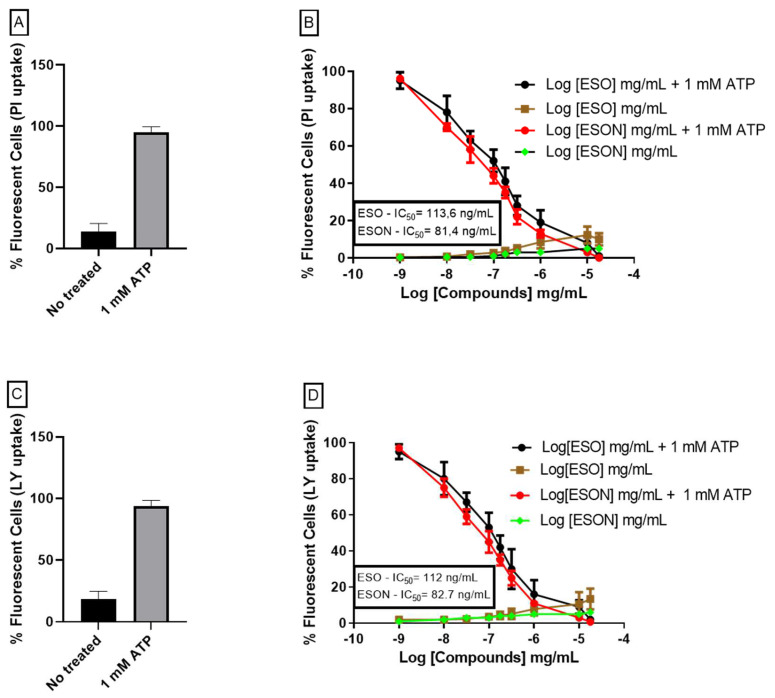
Dose–concentration curves for ESO and ESON inhibition on pore formation activity. (**A**) No treated cells and cells treated with ATP analyzed with PI-uptake assay assay; (**B**) The graph represents the quantification of dye uptake assays performed to 37 °C on MPM. Dose-response curves of ESO and ESON with or without 1 mM ATP for 25 min analyzed by fluorescence plate reader to detect the PI uptake; (**C**) No treated cells and cells treated with ATP analyzed with LY-uptake assay; (**D**) dose–response curves of ESO and ESON with or without 1 mM ATP for 25 min analyzed by fluorescence plate reader to detect the LY uptake. Values represent mean + SEM. Profiles are representative of 3–6 independent experiments.

We induced P2X7R pore formation treating MPM with 1 mM ATP for 25 min. ATP- induced PI (cationic) or LY (anionic) dye uptake was similar [18]. However, ESO treatment inhibited ATP-induced PI and LY uptake with an IC_50_ value of 113. 3 ± 3.7 * ng/mL and 112 ± 2.6 ** ng/mL, respectively (Figure 4A,B). Additionally, the concentrations between 1 and 25 mg/mL spontaneously caused a poor dye uptake (Figure 4A,B). ESON ameliorates the ESO effect, reducing the IC_50_ values for 81.4 ± 7.2 * ng/mL, (* *p* < 0.05) and 82.7 ± 5.5 **, (** *p* < 0.05) ng/mL (Figure 4A,B), respectively. Additionally, ESON did not cause dye uptake when added alone.

### 3.7. ESO and ESON Effect on P2x7r Pore Macroscopic Currents

**Figure 5:**
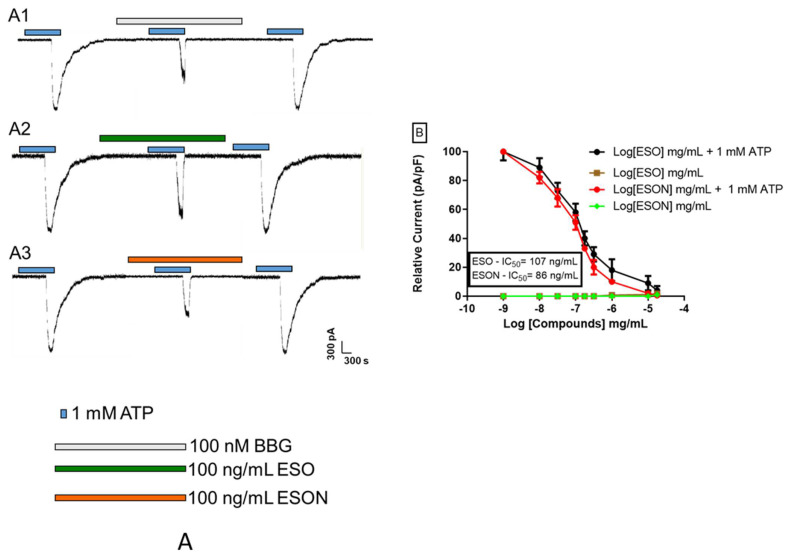
Dose–concentration curves for ESO and ESON inhibition on large-conductance channel activity. (**A**) Whole-cell macroscopic currents recorded P2X7R activated by 1 mM ATP for 5 min at 30–37 °C on MPM. The reversibility of the inhibitory effect of 1 mM ATP for 5 min followed by perfusion of concentrations of ESO, ESON, or 100 nM BBG. After the washout, another pulse of 1 mM ATP was applied; (**B**) the plot represents the quantification of the data observed in (**A1**–**A3**); % relative current recorded as a function of the ratio between the amplitude of the ionic current and cell capacitance. Values represent mean + SEM. Profiles are representative of 4–5 different experiments.

ATP (1 mM) treatment for five min produces macroscopic currents related to P2X7R pore formation (Figure 5A1–A3, first recording). Previous treatment with 100 nM BBG, 100 ng/mL ESO or 100 ng/mL ESON inhibited the pore opening (Figure 5A1–A3, middle recording). All substances exhibited an irreversible antagonist mechanism (Figure 5A1–A3, last recording). Crescent ESO concentrations inhibited the ATP-stimulated large pore current with IC_50_ values of 107 ± 5.2 ng/mL (Figure 5B). ESON reduced ATP-induced macroscopic pore opening currents with IC_50_ values of 86 ± 2.1 * ng/mL (* *p* < 0.05), Figure 5B.

According to these results, the ESO and ESON potently blocked P2X7R large-conductance pore effects and poorly affected the cation channel activity.

### 3.8. ESO and ESON Effect on ATP-Induced IL-1β

MPM excited with LPS (100 ng/mL) for 4 h and stimulated with 1 mM ATP in the final 30 min of LPS incubation evoked IL-1β release. ESO or ESON, when added in the last 60 min of LPS incubation, reverted the ATP effect. ESO inhibited the ATP-induced IL-1β release with an IC_50_ value of 274 ± 91 ng/mL (Table 4). Compared with A74003, ESO exhibited a less potent effect (Table 4). ESON treatment potently reduced the ATP-induced IL-1β release with an IC_50_ value of 62 ± 2 * ng/mL, (* *p* < 0,01) (Table 4).

Another pro-inflammatory cytokine that elicits LPS via Toll-like receptor 4 (TLR4) is the tumor necrosis factor-α (TNF-α). Thus, we evaluated the ESO and ESON inhibitory activity against ATP-induced TNF-α release. ESO inhibited the TNF-α production with an IC_50_ value of 295 ± 8 ng/mL, an effect less than A740003 (Table 4). ESON potently inhibited ATP-induced TNF-α release with a value of 73 ± 9 * ng/mL (* *p* < 0.001), Table 4. ESON ameliorated the ESO effect and potently reduced cytokine levels with potency higher than A740003 (Table 4).

### 3.9. ESO and ESON Effect in Carrageenan-Induced Peritonitis

**Figure 6:**
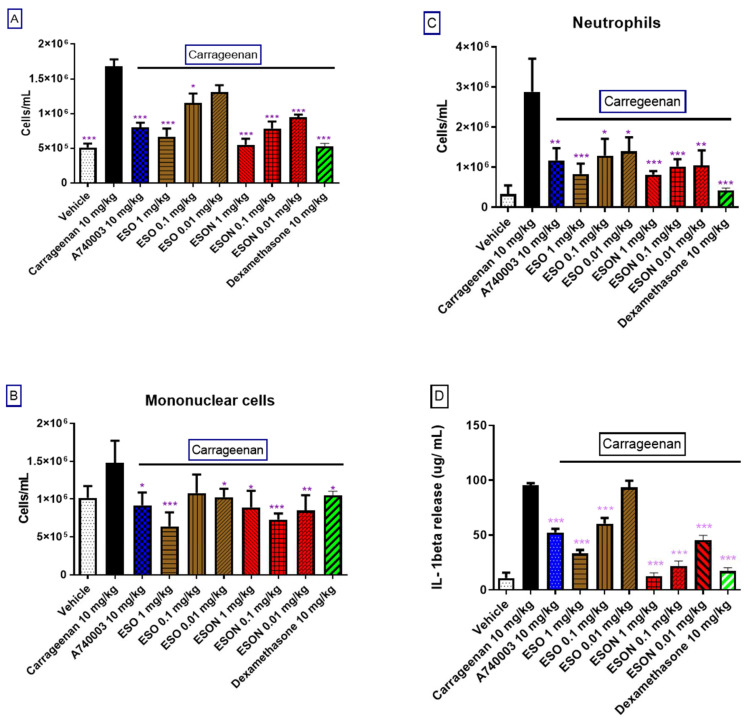
Effect of ESO and ESON on ATP-induced pleurisy. Mice orally pretreated (1 h) with ESO and ESON (1, 0.1, and 0,01 mg/kg), dexamethasone and A740003 (10 mg/kg) before carrageenan-challenge. (**A**) Total cellularity, (**B**) number of mononuclear cells, (**C**) number of neutrophils, and (**D**) level of IL-1β. The results are represented as the means ± SD from 4 assays on different days with five mice for each group. * *p* < 0.05; ** *p* < 0.01; *** *p* < 0.001 in comparison with the carrageenan group.

Carrageenan was applied for 4 h in the peritoneal cavity of mice. Carrageenan administration augmented the IL-1β release in this cavity and the number of mononuclear cells, neutrophils, and eosinophils (Figure 6). Dexamethasone and A740003, in 10 mg/kg, administered 1 h before carrageenan stimulus reversed all these parameters. Both substances administrated alone did not increase these parameters. ESO caused a moderate inhibitory effect for carrageenan-induced cell migration, protein accumulation, and cytokine release (Figure 6B–D). This substance discretely increased the cellularity and protein accumulation when treated alone. In contrast, ESON did not alter the basal levels when administered alone (Supplementary Figure S2).

### 3.10. β-Caryophyllene Activity In Vitro and In Vivo

**Scheme 1:**
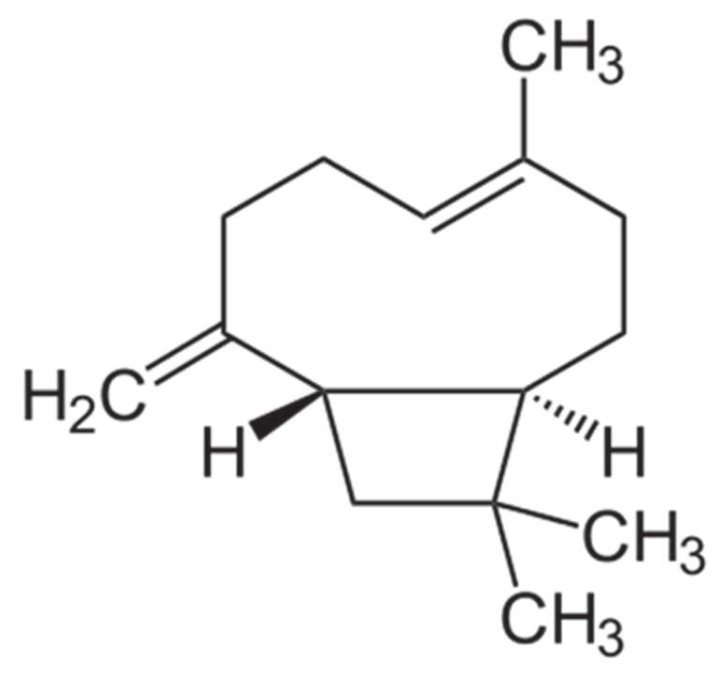
β-caryophyllene 2D structure.

**Figure 7:**
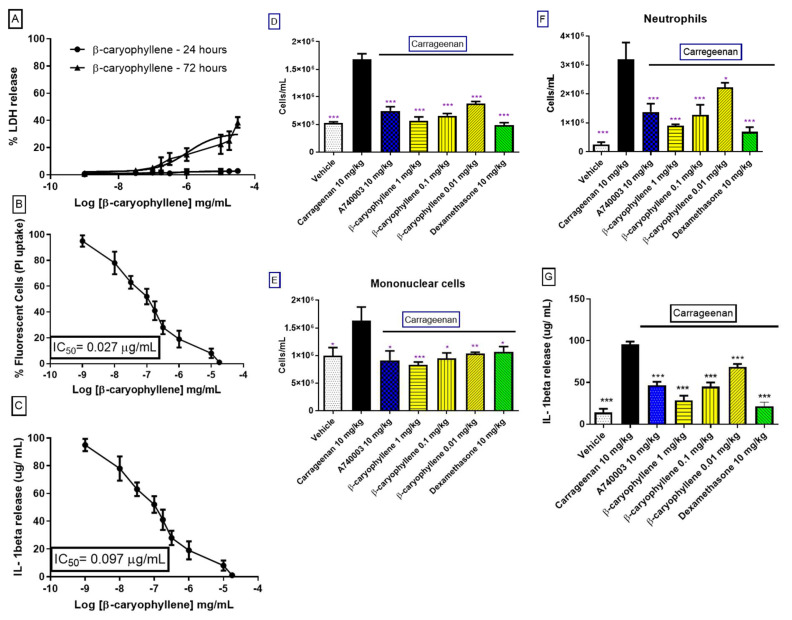
β-caryophyllene effect on P2X7R function and inflammatory response. (**A**) LDH release quantification after the stimulus with crescent β-caryophyllene concentrations for 24 and 72 h; (**B**) The graph represents the quantification of dye uptake assays performed to 37 °C on MPM. Dose response curves of β-caryophyllene concentrations with or without 1 mM ATP for 25 min analyzed, by fluorescence plate reader, to detect the PI uptake; (**C**) Dose –response curves of β-caryophyllene with or without 1 mM ATP for 25 min analyzed by fluorescence plate reader to detect the PI uptake; (**D**) total leukocytes, (**E**) mononuclear cells, (**F**) neutrophils, and (**G**) IL-1β per pool of peritoneal cavity of Swiss webster mice orally pretreated (1 h) with β-caryophyllene (1, 0.1, and 0.01 mg/kg), dexamethasone and A740003 (10 mg/kg), counted under light microscopy 24 h after carrageenan-challenge. Results are expressed as means ± SD from at least 4 experiments on different days with three animals for each group. * *p* < 0.05; ** *p* < 0,01; *** *p* < 0.001 when compared with carrageenan group.

β-caryophyllene is the primary essential oil component (Scheme 1). Thus, we evaluated this molecule as a critical responsibility for inhibiting the P2X7R function. After 24 h of analysis, the dose–response curve promoted moderate toxicity with a CC_50_ value of 29 ± 2 µg/mL (Figure 7A). In the ATP-induced dye uptake assay, β-caryophyllene potently inhibited the P2X7R function with an IC_50_ value of 26 ± 0.007 ng/mL (Figure 7B). Additionally, this substance reduced ATP-induced IL-1β with an IC_50_ value of 97 ± 0.012 ng/mL (Figure 7C). Carrageenan-induced mice peritonitis’ effect was moderately inhibited for 1 µg/kg β-caryophyllene previously treated for 1 h. However, 0.1 and 0.01 µg/kg potently reversed the carrageenan effect comparable to dexamethasone and A740003 treatments (Figure 7D).

### 3.11. β-Caryophyllene Interaction with P2X7R Allosteric Sites

The molecular docking indicated an excellent potential for the β-caryophyllene to bind into the P2X7R. Comparing the binding energy of the 20 conformations generated from the blind molecular docking of both ligands tested (β-caryophyllene and the A740003), the β-caryophyllene presented similar binding energy values (difference less than 1.5 kcal/mol) to the A740003. Table 5 and Table 6 show the energy values and binding sites for each conformation generated by the blind molecular docking for ligands A740003 and β-caryophyllene, respectively.

**Figure 8:**
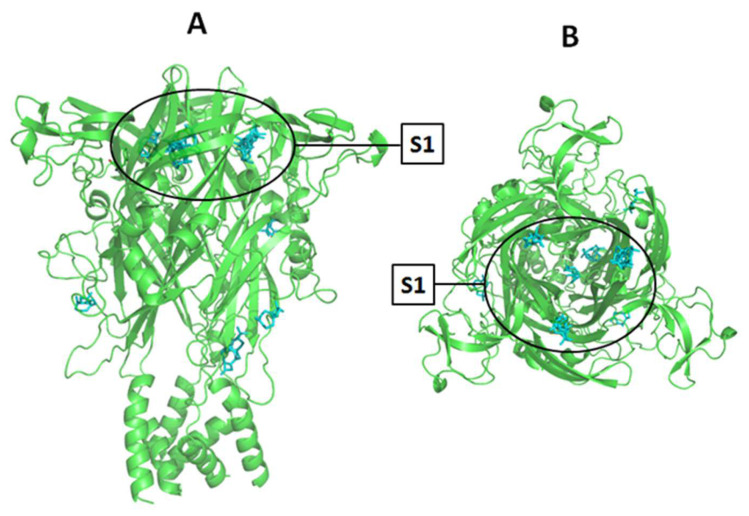
Superposition of all β-caryophyllene conformations generated by the AutoDockVina against the P2X7R macromolecule. The binding site 1 (upper body domain of P2X7R pore) is highlighted and assigned as S1: (**A**) front view, (**B**) top view.

The β-caryophyllene showed an affinity for the same allosteric binding site as the known antagonist, the A740003. This allosteric site is in the upper body domain of the P2X7R pore, comprised of two neighboring subunits. Figure 8 shows the superposition of the β-caryophyllene conformations into the P2X7R. As the P2X7R is a trimeric structure, this receptor presents three similar allosteric binding sites. Therefore, we can note from Figure 2B that the β-caryophyllene conformations are distributed mainly into the three identical allosteric binding sites. 

**Figure 9:**
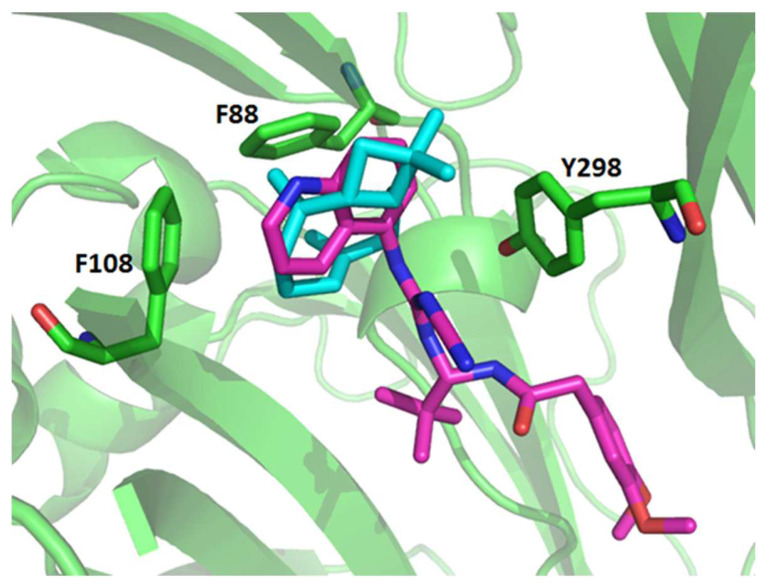
Representation of the possible conformations of the β-caryophyllene docked into the P2X7R. The P2X7 is represented in cartoon and colored in green. The residues from the P2X7R that present the most interaction with the ligand β-caryophyllene are shown in the stick. The β-caryophyllene is depicted in cyan. The ligand A740003 derived from the crystallographic structure (PDB: 5U1U) is depicted in pink.

The conformation with the most favorable binding energy into the allosteric binding pocket is shown in more detail in Figure 9. In the allosteric site, it is possible to note that the β-caryophyllene has a hydrophobic interaction with the lateral chain from the residues Phe88, Phe108, and Try298.

### 3.12. β-Caryophyllene Physical–Chemical Properties

The evaluation of physical–chemical properties indicates that β-caryophyllene presented similar characteristics to the commercial anti-inflammatory drugs (Table 7). Although the β-caryophyllene presented high lipophilicity and low solubility due to the absence of polar heteroatoms in its structure, this substance followed the Lipinski rule of five.

Thus, for the metabolism analysis, we evaluated the interaction of the molecules in question with the main enzymes of the CYP 450 family (1A2, 2C9, 2C19, 2D6, and 3A4), the clearance rate of major CYP enzymes involved in human metabolism, and the excretion facility of glucuronosyltransferase (UGT) activity.

Theoretical results indicate that β-caryophyllene has a lower affinity for plasma proteins than commercial anti-inflammatory drugs for clinical use (Table 8). β-Caryophyllene presented a metabolization profile different from all clinically used anti-inflammatory drugs, mainly metabolized by CYP2C9, CYP2C19, and CYP2D6 (Supplementary Table S1). The intrinsic clearance rate for molecules have been indicated as a substrate for the CYPs (Supplementary Table S2). All molecules analyzed (β-caryophyllene and clinically used anti-inflammatory drugs) inhibit at least one of the CYP enzymes, suggesting that all molecules have a potential for drug interaction since the inhibition of one of the CYP enzymes can affect the metabolism of other drugs by this same enzyme (Supplementary Table S3).

Such characteristics suggest that β-caryophyllene has the potential to become an orally active drug in humans.

### 3.13. β-Caryophyllene Toxicological Profile In Silico

The β-caryophyllene presented a low overall toxicological risk profile, as indicated by the TOX_Risk parameter, a computational filter developed by Simulations Plus using a refined subset of WDI, which includes a diverse set of toxicological models. Also, the β-caryophyllene presented the best result regarding the mutagenic potential compared to the commercial anti-inflammatory drugs (Table 9).

Although β-caryophyllene presented no potential to block the cardiac potassium channel (hERG), this substance presented some hepatotoxicity potential, as it presented alteration in alkaline phosphatase and GGT enzymes (Table 9).

In addition, the β-caryophyllene represented a potential reproductive toxicity risk. This risk is related to anything disturbing the reproductive process of organisms, including adverse effects on sexual organs, behavior, ease of conception, teratogenicity, and developmental toxicity to offspring before or after birth (Table 9).

## 4. Discussion

Lima and colleagues have previously described the chemical composition of the ESO from leaves in 2012 [8] that related the larger fraction of sesquiterpenes (58.2%) to a yield of 1.06%. The major compounds were β-caryophyllene (24.6%) followed by α-pinene (17.2%) and β-pinene (10.9%) [8]. On the other hand, Ramos and collaborators in 2011 [29] described a 0.46% yield to ESO with the largest fraction of monoterpenes (63.1%). The 1,8 cineole (19.0%), α-pinene (16.9%), and β -pinene (14.5%) were the main constituents. In the current study, the sesquiterpenes were a larger fraction in the oil, corroborating with the sesquiterpene fraction [8]. The main compound was β-caryophyllene (18.65%), also described as major by Lima and collaborators [8]. However, Ramos et al. [29] described β-caryophyllene with 5.5%. In addition, the presence of α-pinene (6.75%) and β-pinene (4.0%) in major quantities in the actual study corroborates with the reports from both Lima et al. and Ramos et al., suggesting an important role of these monoterpenes in the essential oil chemical profile of leaves from the *E. sulcata* [8,29]. Lastly, chemical variations in the composition or yield of a plant species’ essential oil are widely described in the literature due to the adaptability and modulation of the secondary metabolism in response to different environmental conditions [30].

We prepared the nanoformulation to enable the administration of the essential oil of *E. sulcata* by different routes and allow nanoformulation use in aqueous matrices, since essential oils have non-polar characteristics that lead to phase separation. The low energy method by phase inversion with temperature was selected for not using organic solvents and allowing the reproducibility to industrial scale [31]. Among all surfactants’ volumes presented in Table 2, the formulations with HLB between 15.5 and 16.7 can be classified as nanoemulsions concerning their average droplet size between 20–200 nm [32]. In addition, they also showed a translucent bluish aspect, characteristic of the Tyndall effect in nanoemulsified systems [33]. The HLB that showed the highest affinity with ESO was 16.25, which resulted in the smallest of 132.83 ± 3.12, indicating ESO has hydrophilic properties [34]. The correct selection of surfactants is a critical step in formulating a nanoemulsion. This formulation forms the interface between the oil and aqueous phases of the system, directly affecting the formation of droplets and their stability [35]. In addition, our group refined the nanoemulsion (Table 3). Another 10 formulations were prepared, varying the amounts (2.5–7.5%) of essential oil and the mixture of surfactants with HLB 16.25. Formulation F6 was selected as the most promising, exhibiting an average droplet size of 76.57 ± 4.32 and 0.438 ± 0.02 PDI, considering it had a small particle size (76.57 ± 4.32) and used the least number of surfactants in the formulation (5%). F6 also had the lowest amount of surfactants blend (5%) between the nanoemulsions with a mean size above 200 nm. F8 and F9 presented lower droplet size values. However, they had higher concentrations of surfactants (7.5% and 10%, respectively) and consequently higher toxicity leading to the selection of F6 as the optimal ESON.

Essential oils have been considered harmful in the eucaryotic life form. Thus, their application should undergo a toxicological study [34]. Their biological results depend on their chemical composition. Essential oils are modified by their degradation rate and bioavailability in the gastrointestinal portion of organisms. The digestive system diminishes its effectiveness and raise the doses administered [35]. One of the appropriate alternatives to conserving essential oils from degradation is encapsulation into microparticles or nanoparticles [36].

ESO promoted moderate toxicity on MPMs in vitro after 72 h of continuous stimulus. Therefore, we tested ESON, which possess higher dispersibility in an aqueous medium than ESO. As expected, ESON reversed ESO toxicity. In this context, water-in-oil-in-water (W/O/W) nanoemulsions consisting of surfactants with a variety of Polysorbate-85/Labrasol^®^, Polysorbate-85/Cremophor^®^ EL, or glycerol/Polysorbate-85 were checked concerning cytotoxicity against mammalian cells. Labrasol and Cremophor induced apoptotic death, chromatin condensation, and P2X7R cell death activation. However, the nanoemulsion containing glycerol was not harmful [37].

For an already ESON-ameliorated ESO toxicity profile, we tested the inhibitory activity of both compounds on P2X7R function. ATP-induced P2X7R ion channel was not reversed by ESO, and was reversed by ESON. In contrast, ATP-induced pore formation was impaired for ESO and ESON. Furthermore, ESON prominently diminished the ATP-induced dye uptake with an effect higher than ESO.

Additionally, both compounds reduced ATP-induced IL-1β release, and ESON showed inhibitory activity higher than ESO. This potential selectivity for inhibiting the P2X7R pore is extremely relevant to discriminate the intracellular pathway and functions associated with large conductance pore pathway and ionic channel activity [18]. Thus, the ESO and ESON selectivity may represent a significant tool for studying the P2X7R channel activity in physiological and pathological conditions.

Other essential oil nanoemulsions from plants of the Myrtaceae family, although exhibiting anti-inflammatory activity, in general, are unknown regarding the plasma membrane receptor associated with the intracellular signaling pathway to cause inflammation reduction. Clove essential oil (CO) is extracted from the flower buds of *Syzygium aromaticum* (*Eugenia caryophyllata* L., Myrtaceae). CO and their encapsulation into nanoemulsion were investigated concerning their wound-healing effects. Their nanoemulsion exhibited significant healing effects in rats and an enhancement in leucine content compared to pure CO. Rats treated with nanoemulsion did not present signs of inflammatory cells in the histopathological analysis [38]. *Eucalyptus globulus* is an aromatic medicinal plant from the Myrtaceae family containing 1,8-cineole as the major pharmacological constituent. An *E. globulus*-loaded micellar nanoparticle was developed for evaluating the analgesic efficacy in rats. The transdermal administration of micellar nanoparticles of *Eucalyptus globulus* on rats’ fore and hind limbs prolonged the central and peripheral analgesic effects [39].

Essential oils from other plant families can also reverse inflammation. Coincidently, possible targets in the plasma membrane for these compounds are scarce.

*Rosmarinus officinalis* L. (OERO) essential oil possesses anti-inflammatory activity. Thus, the anti-inflammatory potency of OERO nanoemulsions (NOERO, NECHA, NECULT, and NECOM) were tested in vitro and in vivo. In addition, the cellular antioxidant activity (CCA), nitric oxide production, cellular viability, and anti-inflammatory activity were evaluated in zebrafish. All nanoemulsions demonstrated an absence of cytotoxicity, antioxidant activity, and a potentiation of the OERO effect. Additionally, nanoemulsions potentiated the OERO anti-inflammatory action reducing pro-inflammatory mediator production in zebrafish [40].

Carvacrol (CV) is an essential oil with immunomodulatory activity. Thus, a carvacrol-loaded nanoemulsion (CVNE) nanoemulsion was used for evaluating their immunomodulatory action. CV did not exhibit cytotoxicity and reduced the production of IL-2 in the peripheral blood mononuclear cell (PBMC) culture supernatants. CVNE showed no cytotoxicity and impaired IL-2, IL-17, and IFN-γ levels [41]. Essential oils from *Rimulus cinnamon* (EORC) decreased the numbers of neutrophils and nitric oxide (NO) levels in the lung. Additionally, EORC reduced the mRNA expression of pyrin domain-containing 3 (NLRP3), IL-1β, nitric oxide synthase (iNOS), and the protein expression of NLRP3, caspase-1 (p20), pro-IL-1β, and P2X7R in the lung tissues [42]. Thus, this study indicated a molecular target in the plasma membrane possibly associated with essential oil anti-inflammatory effects. Additionally, essential oil inclusion in a nanoemulsion potentializes the essential oil effects.

Mice pretreated with ESO reduced carraggeenan-induced inflammation. When individually applied, the essential oil generated a modest inflammatory reaction, a result that can be considered toxic. ESON pretreatment inhibited carrageenan-induced inflammation and did not evoke cellular migration. Thus, the nanoformulation potentiated the P2X7R inhibition in vitro and the inflammatory response inhibition in vivo.

The β-caryophyllene was identified as the main substance from ESO. Thus, we investigated the inhibitory effect of this substance against P2X7R function. β-caryophyllene reproduced the profile observed after ESON treatment for inhibiting the P2X7R function in vitro and the inflammatory response in vivo. Interestingly, when we evaluated the β-caryophyllene interaction with P2X7R, the residues were the same as previously reported to interact with the known allosteric ligand A740003 [27]. Therefore, β-caryophyllene might present a similar mechanism of P2X7R inhibition as the A740003 ligand.

Additionally, β-caryophyllene demonstrated potency, efficacy, and action mechanism similar to that previously described for physalins [28]. The crude extract from *Physalis angulata* and the pool of isolated physalins (B, D, F, and G) impaired P2X7R function in vitro. Physalin D, in contrast to physalin B, F, and G, inhibited ATP-induced paw edema, ATP, and lipopolysaccharide (LPS)-induced pleurisy. Molecular modeling and computational predictions determined physalin D and F as potent allosteric P2X7R antagonists [28].

Other isolated natural molecules also inhibited P2X7R activity in vitro, albeit not presenting data about molecular binding with P2X7R in silico. Fischer and collaborators appointed teniposide as a P2X7R inhibitor in vitro [43]. Stylissadines A and B, two Australian marine sponges from *Stylissa flabellate*, reversed P2X7R-dependent dye uptake [44]. Rhein (4, 5-dihydroxyanthraquinone-2-carboxylic acid), a rhizome of rhubarb, inhibited rat P2X7R function in micromolar concentrations [45]. Emodin (1,3,8-trihydroxy-6-methylanthraquinone), an anthraquinone analog isolated from *Rheum officinale* Baill, reversed pore formation activity [46] and IL-1β release [47] in micromolar concentrations. The inhibitory response for β-caryophyllene was higher than observed for teniposide, stylissadines, Rhein, and emodin, even though physalin D showed an inhibitory profile similar to β-caryophyllene.

Furthermore, we analyze the metabolic and plasma protein binding parameters for these molecules compared to clinically used anti-inflammatory drugs. Most drug metabolism (~75%) occurs through reactions catalyzed by the CYP 450 family enzymes, distributed in several tissues, mainly in the liver, intestinal tract, and kidney. CYP 450 enzymes act, in general, through the oxidation of substrates, which can lead to their inactivation or activation (in the case of pro-drugs). Furthermore, the drug can also inhibit CYP, which is generally associated with drug interactions [48]. Thus, β-caryophyllene presented a metabolization profile different from all clinically used anti-inflammatory drugs, a factor that is possibly positive for this substance. Additionally, β-caryophyllene tends to have drug interactions, especially with drugs that also bind plasma proteins with a high fraction. However, its free amount is superior to commercial anti-inflammatory drugs.

β-Caryophyllene has no site for the glucuronidation reaction by the UGT enzyme since the glucuronidation reaction occurs in molecules that have at least one nucleophilic group [48]. As β-caryophyllene has only carbons in its constitution, the ADMET program did not assess its propensity for killing by the UGT (data not shown). Additionally, this molecule does not go through the elimination route by the UGT enzyme as it does not present a site for the glucuronidation reaction. The physical–chemical analysis suggests that β-caryophyllene has the potential to become an orally active drug in humans, and the toxicological profile suggests that β-caryophyllene is predicted to have a low overall toxicological risk profile. Additionally, β-caryophyllene represented a potential reproductive toxicity risk.

## 5. Conclusions

The essential oil from *Eugenia sulcata* leaves, ESO, inhibited P2X7R activity in vitro and the inflammatory response in vivo. However, ESO caused moderated toxicity in vitro and recruited neutrophils in mice. ESON was not toxic in vitro and in vivo, did not augment the cellularity in mice, and was more effective than ESO for inhibiting the P2X7R function and inflammation in vivo. The ESO principal substance, β-caryophyllene, inhibited P2X7R function in vitro and the inflammation in vivo similar to ESON. This substance binds to P2X7R allosteric situs, reducing their function. In silico analysis indicates β-caryophyllene as a candidate for oral administration with reduced toxicological risk. Therefore, ESO effects potentialized and ameliorated for ESON can be reproduced for isolated substance β-caryophyllene to inhibit P2X7R function and the inflammatory response. The essential oil nanoformulation improved the potency to inhibit P2X7R in vitro and inflammation in mice. Thus, ESO nanoformulation is a good pharmacological strategy for investigating the P2X7R-mediated inflammatory response in physiological or pathological conditions.

## Figures and Tables

**Table 1:** Chemical composition of essential oil of *Eugenia sulcata* fresh leaves after search by GC-FID and GC-MS.

Peak	RT	IA	IA exp	Substances	%
2	5.165	933	932	α-Pinene	6.75
4	6.231	977	974	β-Pinene	4.00
9	7.724	1028	1025	Sylvestrene	1.02
10	7.802	1030	1026	1,8-Cineole	3.21
18	13.674	1191	1186	α-Terpineol	0.98
20	19.212	1326	1324	Myrtenyl acetate	0.44
22	20.200	1350	1348	α-Cubebene	1.29
24	21.088	1371	1373	α-Ylangene	0.41
25	21.280	1376	1374	α-Copaene	3.22
27	21.636	1385	1387	β-Bourbonene	0.40
29	21.941	1392	1389	β-Elemene	0.81
31	22.652	1410	1409	α-Gurjunene	0.59
32	23.150	1422	1417	β-Caryophyllene	18.65
33	23.434	1429	1430	β-Copaene	0.88
34	23.548	1432	1431	β-Gurjunene	0.85
37	23.811	1439	1439	Aromadendrene	0.41
40	24.287	1451	1451	trans-Muurola-3,5-diene	1.00
41	24.422	1454	1452	α-Humulene	5.61
43	24.685	1461	1458	allo-Aromadendrene	1.07
44	25.027	1469	1471	Dauca-5,8-diene	1.17
45	25.204	1474	1475	trans-Cadina-1(6),4-diene	0.89
46	25.347	1477	1478	γ-Muurolene	2.56
47	25.489	1481	1480	Germacrene D	1.07
48	25.695	1486	1489	β-Selinene	3.17
49	25.908	1491	1493	trans-Muurola-4(14),5-diene	0.41
50	26.057	1495	1498	α-Selinene	3.17
51	26.271	1501	1500	α-Muurolene	1.16
52	26.605	1509	1506	(Z)-α-Bisabolene	0.92
53	26.797	1514	1513	γ-Cadinene	1.75
54	27.195	1525	1522	δ-Cadinene	10.02
55	27.529	1534	1533	trans-Cadina-1,4-diene	4.63
56	27.707	1538	1537	α-Cadinene	0.40
58	27.891	1543	1544	α-Calacorene	0.57
67	29.398	1583	1582	Caryophyllene oxide	2.17
68	29.910	1596	1594	Carotol	1.79
69	30.159	1603	1602	Ledol	0.77
77	31.105	1629	1627	1-epi-Cubenol	1.29
81	34.616	1727	1725	Guaiol acetate	1.33
84	32.057	1655	1652	α-Cadinol	1.29
Monoterpene hydrocarbons					11.77
Oxygenated monoterpenes					4.63
Total monoterpenes					16.40
Sesquiterpene hydrocarbons					65.99
Oxygenated sesquiterpenes					9.17
Total sesquiterpenes					75.16
Total identified					91.56

RT–Retention Time; IA–Arithmetic Index; IA exp–Experimental Arithmetic Index.

**Table 2:** HLB range values, the proportion of surfactants, and mean size of emulsions with 5% (*w*/*w*) of *E. sulcata* essential oil.

HLB	Polysorbate 20 (%)	Sorbitan Monooleate 80 (%)	Droplets Size (nm)
12	62.10	37.90	273.93 ± 6.52
13	70.16	29.84	450.56 ± 8.45
14	78.22	21.78	222.31 ± 4.55
15	86.29	13.71	234.23 ± 3.78
15.5	90.32	9.68	134.82 ± 2.17
16	94.35	5.65	192.31 ± 5.37
16.25	96.37	3.63	132.83 ± 3.12
16.5	98.38	1.62	151.10 ± 3.35
16.7	100.0	0.0	199.03 ± 1.07

HBL: hydrophilic–lipophilic balance.

**Table 3:** Arrangement of the optimization of the ESON with the amount of ESO, surfactants blend, water, droplets size, and PdI values.

Formulation	Droplets Size (nm)	PdI	Essential Oil (%)	Surfactants (%)	Water (%)
F1	397.60 ± 10.54	0.925 ± 0.05	2.5	7.5	90.0
F2	179.92 ± 9.31	0.560 ± 0.02	5.0	7.5	87.5
F3	286.43 ± 3.90	0.910 ± 0.03	7.5	5.0	87.5
F4	277.52 ± 4.53	0.527 ± 0.06	7.5	2.5	90.0
F5	246.10 ± 6.14	0.520 ± 0.05	5.0	2.5	92.5
F6	76.57 ± 4.32	0.438 ± 0.02	2.5	5.0	92.5
F7	144.93 ± 7.54	0.383 ± 0.03	5.0	10.0	85.0
F8	46.34 ± 5.63	0.481 ± 0.02	7.5	7.5	85.0
F9	27.15 ± 10.16	0.410 ± 0.03	7.5	10.0	82.5
F10	108.53 ± 8.21	0.412 ± 0.04	5.0	5.0	90.0

HBL: hydrophilic–lipophilic balance; PdI: Polydispersity index.

**Table 4:** ESO and ESON inhibited the ATP-induced cytokines in mouse cells.

Antagonists	IC_50_ Value (µg/mL) ^#^ IL-1β Release	IC_50_ Value (µg/mL) ^#^ TNF-α Release
A740003	0.094 ± 0.009	0.097 ± 0.005
ESO	0.274 ± 0.091 *^,&^	0.295 ± 0.008 ^%,@^
ESON	0.062 ± 0.002 **	0.073 ± 0.009 ^%%^

^#^ All results represent 3–4 experiments in triplicates on three distinct days and are expressed as mean ± SD; * *p* < 0.05 compared with A740003; ^&^ *p* < 0.001 compared with ESON; ** *p* < 0.01 compared with A740003; ^%^ *p* < 0.05 compared with A740003; ^@^ *p* < 0.001 compared with ESON; ^%%^ *p* < 0.01 compared with A740003.

**Table 5:** Binding energy values for each conformation generated by the blind molecular docking for the A740003 (known antagonist). In addition, a reference in this work, the A740003 allosteric site is named site 1, and the ATP binding site is named site 2. A few confirmations were docked in random non-pocket positions in the P2X7R, which were not considered a potential binding site.

Conformation	Energy kcal/mol	Binding Site
1	−10.4	1
2	−9.8	1
3	−9.5	1
4	−9.5	1
5	−8.7	1
6	−8.4	1
7	−8.3	1
8	−8.2	1
9	−8.1	-
10	−8.1	1
11	−8	1
12	−8	-
13	−8	1
14	−8	1
15	−8	1
16	−7.9	1
17	−7.9	1
18	−7.8	2
19	−7.8	1
20	−7.7	1

**Table 6:** Binding energy values for each conformation generated by the blind molecular docking for the β-caryophyllene. As a reference in this work, the A740003 allosteric site is named site 1, and the ATP binding site is named site 2. In addition, a few confirmations were docked in aleatory non-pocket positions in the P2XR, which were not considered a potential binding site.

Conformation	Energy kcal/mol	Binding Site
1	−8.9	1
2	−8.9	1
3	−7.9	1
4	−7.6	-
5	−7.2	1
6	−7.0	-
7	−7.0	1
8	−6.8	1
9	−6.8	1
10	−6.8	1
11	−6.7	1
12	−6.7	1
13	−6.7	-
14	−6.7	-
15	−6.6	1
16	−6.6	-
17	−6.4	-
18	−6.4	1
19	−6.4	1
20	6.3	-

**Table 7:** Physical–chemical parameters of β-caryophyllene compared to commercial anti-inflammatories drugs (diclofenac, ibuprofen, and naproxen). ^a^ Molecular weight (Da); ^b^ Partition coefficient in a logarithmic scale; ^c^ Native water solubility (mg/mL); ^d^ Number of hydrogen bond acceptor (HBA); ^e^ Number of hydrogen bond donor (HBD); ^f^ Number of Lipinski’s rules broken (NLRB).

Compounds	Physical–Chemical Properties					
	MW ^a^	LogP ^b^	S + WS ^c^	HBA ^d^	HBD ^e^	NLRB ^f^
β-cariophyllene	204	4.6	0.002	0	0	0
diclofenac	295	3.89	0.0464	3	2	0
Ibuprofen	206	3.0	0.1	2	1	0
Naproxen	230	2.69	0.0649	3	1	0

**Table 8:** Unbound percentage of the molecules in question with blood plasma proteins.

Molecules	Percentage (%)
β-caryophyllene	5.37
Diclofenac	0.38
Ibuprofen	2.1
Naproxen	1.33

**Table 9:** Toxicological profile of β-caryophyllene comparing to commercial anti-inflammatories drugs (diclofenac, ibuprofen, and naproxen); ^a^ Qualitative estimation of the likelihood of the hERG potassium channel inhibition in human (hERG); ^b^ Qualitative estimation of triggering the mutagenic chromosomal aberrations (CABR); ^c^ human liver adverse effect as the likelihood of causing an elevation in one of the following enzymes: alkaline phosphatase, GGT, and LDH; ^d^ qualitative estimation of reproductive toxicity; ^e^ represents the risk of mutagenicity assessed by ten models of “virtual Ames testing.” (MUT_Risk); ^f^ represents the overall toxicological risk (TOX_Risk).

Compound	Toxicological Profile							
	^a^ hERG	^b^ CABR	^c^ Hepatotoxicity			^d^ Repro	^e^ MUT_Risk	^f^ TOX_Risk
			AlkPhos	GGT	LDH			
β-caryophyllene	No	Nontoxic	Elevated	Normal	Elevated	Toxic	0	1
Diclofenac	Yes	Nontoxic	Elevated	Elevated	Normal	Nontoxic	0.6	2
Ibuprofen	No	Nontoxic	Normal	Normal	Normal	Nontoxic	0.6	0
Naproxen	No	Toxic	Normal	Normal	Normal	Nontoxic	1.2	0

## Data Availability

The data presented in this study are available on request from the corresponding author. The data are not publicly available due to INPI politics related to patented data.

## Supplementary Materials

The following supporting information can be downloaded at: https://www.mdpi.com/article/10.3390/pharmaceutics14050911/s1, Figure S1: Superposition of the A740003 conformation extracted from the PDB ID: 5U1U (depicted in pink) and the most favorable conformation generated by the blind molecular docking (depicted in grey). The P2X7 receptor is depicted in the green cartoon, and the primary residues that showed interaction with the ligand are depicted in the green stick, Figure S2: Substance (A) Total leukocytes, (B) mononuclear cells, and (C) neutrophils per pool of peritoneal cavity of Swiss webster mice orally pretreated (1 hour) with ESO, ESON or β-caryo- phyllene (1 mg/kg), dexamethasone, and A740003 (10 mg/kg), counted under light microscopy 24 h after carrageenan-challenge. Results are expressed as means ± s.d from at least 4 experiments on different days with three animals for each group. * = ESO, ESON and β-caryophyllene effect on mice peritoneal cavity Table S1: Qualitative assessment of the molecules in question being the substrate of CYPs; Table S2: Intrinsic clearance rate (μL/min/mg) for the molecules in question concerning CYP enzymes. The higher the value, the higher the substrate depuration rate; Table S3: A qualitative estimate of the inhibitory action of the molecules in question against CYP enzymes.
