# Supplementary material for: Eugenia sulcata (Myrtaceae) Nanoemulsion Enhances the Inhibitory Activity of the Essential Oil on P2X7R and Inflammatory Response In Vivo
Source: Pharmaceutics. 2022 Apr 21;14(5):911. doi: 10.3390/pharmaceutics14050911 (PMC9148016; doi:10.3390/pharmaceutics14050911)
Supplement: Supplementary file 1 [file pharmaceutics-14-00911-s001.zip › pharmaceutics-1472590-supplementary.pdf]

pharmaceutics

MDPI

Supplementary File

Eugenia sulcata (Myrtaceae) Nanoemulsion Enhances the Inhibitory Activity of the Essential Oil on P2X7R and Inflammatory Response In Vivo

Bettina Quintanilha. Magalhães, Francisco P. Machado, Paola S. Sanches, Bárbara Lima, Deborah Quintanilha. Falcão, Natalia von Ranke, Murilo Lamim Bello, Carlos Rangel. Rodrigues, Marcelo Guerra. Santos, Leandro Rocha and Robson X. Faria

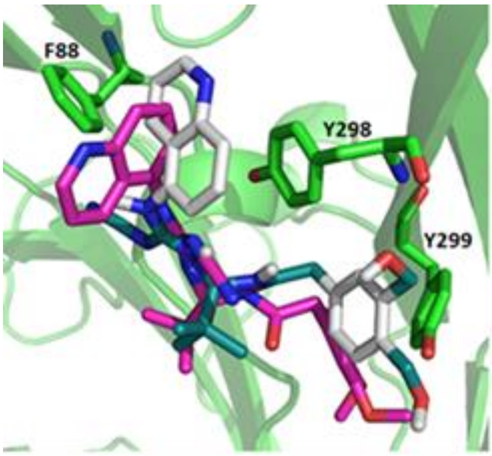

Figure S1. Superposition of the A740003 conformation extracted from the PDB ID: 5U1U (depicted in pink) and the most favorable conformation generated by the blind molecular docking (depicted in grey). The P2X7 receptor is depicted in the green cartoon, and the primary residues that showed interaction with the ligand are depicted in the green stick.

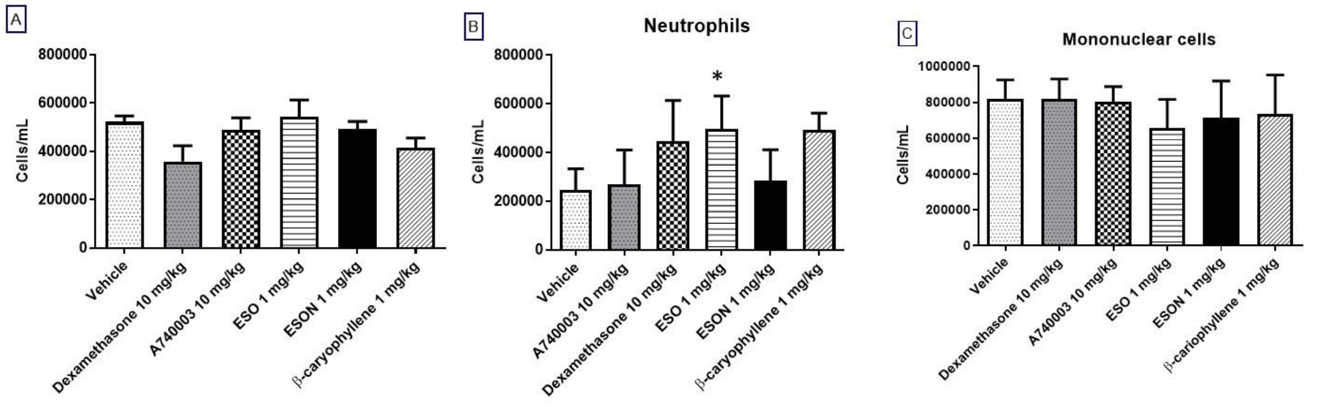

Figure S2. Substance (A) Total leukocytes, (B) mononuclear cells, and (C) neutrophils per pool of peritoneal cavity of Swiss webster mice orally pretreated (1 hour) with ESO, ESON or β-caryophyllene (1 mg/kg), dexamethasone, and A740003 (10 mg/kg), counted under light microscopy 24 h after carrageenan-challenge. Results are expressed as means ± s.d from at least 4 experiments on different days with three animals for each group. * = ESO, ESON and β-caryophyllene effect on mice peritoneal cavity.

Table S1. Qualitative assessment of the molecules in question being the substrate of CYPs. .

pharmaceutics

MDPI

Molecules	CYP1A2	CYP2C9	CYP2C19	CYP2D6	CYP3A4
β-caryophyllene	NO	NO	YES	NO	YES
Diclofenac	NO	YES	YES	NO	NO
Ibuprofen	NO	YES	YES	YES	NO
Naproxen	NO	YES	YES	YES	NO

Table S2. Intrinsic clearance rate (μL/min/mg) for the molecules in question concerning CYP enzymes. The higher the value, the higher the substrate depuration rate.

Molecules	CYP1A2	CYP2C9	CYP2C19	CYP2D6	CYP3A4
β-caryophyllene	-	-	1.74	-	22.7
Diclofenac	-	78.1	0.282	-	-
Ibuprofen	-	89.3	2.39	18.7	-
Naproxen	-	28.5	0.0755	0.23	-

Table S3. A qualitative estimate of the inhibitory action of the molecules in question against CYP enzymes.

Molecules	CYP1A2	CYP2C9	CYP2C19	CYP2D6	CYP3A4
β-caryophyllene	NO	NO	YES	NO	NO
Diclofenac	NO	YES	NO	NO	NO
Ibuprofen	NO	YES	NO	NO	NO
Naproxen	NO	YES	NO	NO	NO
